# A computerised decision support system for cardiovascular risk management ‘live’ in the electronic health record environment: development, validation and implementation—the Utrecht Cardiovascular Cohort Initiative

**DOI:** 10.1007/s12471-019-01308-w

**Published:** 2019-08-01

**Authors:** T. K. J. Groenhof, Z. H. Rittersma, M. L. Bots, M. Brandjes, J. J. L. Jacobs, D. E. Grobbee, W. W. van Solinge, F. L. J. Visseren, S. Haitjema, F. W. Asselbergs, Members of the UCC-CVRM Study Group, Pim A. de Jong, Marianne C. Verhaar, Frank L. J. Visseren, Folkert W. Asselbergs, Niels P. van der Kaaij, Imo E. Höfer, Gert-Jan de Borst, Ynte M. Ruigrok, Monika Hollander, Stefan M. Dieleman, A. Titia Lely, Mariëlle H. Emmelot-Vonk, Michiel L. Bots

**Affiliations:** 1 Julius Centre for Health Sciences and Primary Care, University Medical Centre Utrecht, Utrecht University, Utrecht, The Netherlands; 2 Department of Cardiology, Division Heart and Lungs, University Medical Centre Utrecht, Utrecht University, Utrecht, The Netherlands; 3 ORTEC B.V., Zoetermeer, The Netherlands; 4 Department of Clinical Chemistry and Haematology, University Medical Centre Utrecht, Utrecht University, Utrecht, The Netherlands; 5 Department of Vascular Medicine, University Medical Centre Utrecht, Utrecht University, Utrecht, The Netherlands; 6 Institute of Cardiovascular Science, Faculty of Population Health Sciences, University College London, London, UK; 7 Health Data Research UK and Institute of Health Informatics, University College London, London, UK

**Keywords:** Computerised decision support system, Cardiovascular risk management, Adherence, Real-world data, Big data, Health information technology

## Abstract

**Purpose:**

We set out to develop a real-time computerised decision support system (CDSS) embedded in the electronic health record (EHR) with information on risk factors, estimated risk, and guideline-based advice on treatment strategy in order to improve adherence to cardiovascular risk management (CVRM) guidelines with the ultimate aim of improving patient healthcare.

**Methods:**

We defined a project plan including the scope and requirements, infrastructure and interface, data quality and study population, validation and evaluation of the CDSS.

**Results:**

In collaboration with clinicians, data scientists, epidemiologists, ICT architects, and user experience and interface designers we developed a CDSS that provides ‘live’ information on CVRM within the environment of the EHR. The CDSS provides information on cardiovascular risk factors (age, sex, medical and family history, smoking, blood pressure, lipids, kidney function, and glucose intolerance measurements), estimated 10-year cardiovascular risk, guideline-compliant suggestions for both pharmacological and non-pharmacological treatment to optimise risk factors, and an estimate on the change in 10-year risk of cardiovascular disease if treatment goals are adhered to. Our pilot study identified a number of issues that needed to be addressed, such as missing data, rules and regulations, privacy, and patient participation.

**Conclusion:**

Development of a CDSS is complex and requires a multidisciplinary approach. We identified opportunities and challenges in our project developing a CDSS aimed at improving adherence to CVRM guidelines. The regulatory environment, including guidance on scientific evaluation, legislation, and privacy issues needs to evolve within this emerging field of eHealth.

## What’s new?

- Adherence to guidelines may improve if these are shown to patients and their healthcare providers in an electronic health record.
- Clinical decision support tools that generate patient-specific assessments or recommendations may improve practitioner performance without additional utilisation of healthcare resources if data are collected in a standardised manner.
- Guidance on scientific evaluation of eHealth tools is much needed to determine whether implementation cost-effectively improves patient outcomes.

## Introduction

Modification of vascular risk factors is effective in reducing cardiovascular morbidity and mortality in patients with a cardiovascular condition [1]. Cardiovascular management guidelines provide various recommendations for modification [2]. Yet, there is a gap between guidelines and practice: adherence to guidelines varies between medical disciplines and between treating physicians, even for similar patients [3]. This leads to suboptimal risk factor screening and management, which eventually leads to preventable excess morbidity and mortality [4].

Studies suggest that adherence to guidelines improves if attention is drawn to them, for instance by presenting clinicians with the latest guideline evidence together with individual patient information ‘live’ in the electronic health record (EHR) in an easy accessible online format [5, 6]. This type of ‘live’ computerised decision support system (CDSS) is an electronic system designed to aid clinical decision-making by generating patient-specific assessments or recommendations that are presented to clinicians. Also, CDSSs can support benchmarking by detecting cases where suboptimal decisions were made—i.e. decisions that did not conform to the latest guidelines. As a new component of the clinical infrastructure, CDSSs may improve processes and practitioner performance without additional utilisation of healthcare resources, and ultimately results in improvement of patient outcomes [5, 7].

Here we describe the development and pilot findings of such a CDSS.

## Methods

### Incentive, scope, requirements, and clinical content

Development of the CDSS started with defining the project and its incentive. It is well established that CDSSs developed together with clinicians are used more frequently than those developed by industry alone [6, 8]. We created an expert group comprising clinicians, data scientists, epidemiologists, ICT architects, and user experience and user interface designers. This expert group defined the scope of our pilot as a ‘cardiovascular risk management (CVRM) dashboard in the EHR environment of the University Medical Centre Utrecht, scalable to other types of EHR for use in routine clinical practice and accessible to patients’.

A project plan was drafted defining aims on timing, content, and functionalities. The CVRM dashboard should provide the treating physician with ‘live’ information in the EHR environment during the patient visit. The content comprised cardiovascular risk factors (age, sex, medical and family history, smoking, blood pressure, lipids, kidney function, glucose intolerance), estimated 10-year cardiovascular risk, guideline-compliant strategies for (non-)pharmacological treatment to optimise risk factors, and an estimated change in 10-year risk of cardiovascular disease (CVD) if treatment goals are adhered to [1, 9]. It was requested that treatment benefit be incorporated into the risk calculation in such a way that clinicians and patients could switch these prospects ‘on’ and ‘off’ to explore the effects of certain interventions. Risk assessment for CVD is calculated differently for patients with different (co-)morbidities. These risk factors, scores, and guideline-compliant strategies together formed a decision tree with business rules. In these business rules we determine which risk assessment methodology to use for patients with different co-morbidities, such as (1) with no previous cardiovascular events, HEART score; (2) with a history of one or more cardiovascular events, SMART score; (3) with diabetes, ADVANCE; and (4) elderly patients, Elderly score. Each of these methodologies defines treatment targets, thus suggestions for therapy can be generated from the profile and risk score combined. The decision tree was derived from the current cardiovascular guidelines. Possible conflicts were discussed until a consensus was reached in an expert group comprising 12 cardiovascular specialists.

Administrative support was requested, integrating a print function and a copy-to-consult function to the CDSS. Direct links to the manuscripts and guidelines were added to allow physicians easy access to sources of evidence for risk scores and treatment recommendations. Lastly, the CDSS had to be accessible via patient portals.

### Infrastructure and interface

The technical framework of a CDSS scopes all technical aspects from data collection (and storage) to user interface. This technical blueprint comprises information on data location, location and functionalities of the final application, and connections and steps in-between. In our project, important criteria for the selection of a technical structure were (1) visualising the CDSS within the EHR and (2) sustaining scalability. Therefore, we chose a web-based application.

**Fig. 1:**
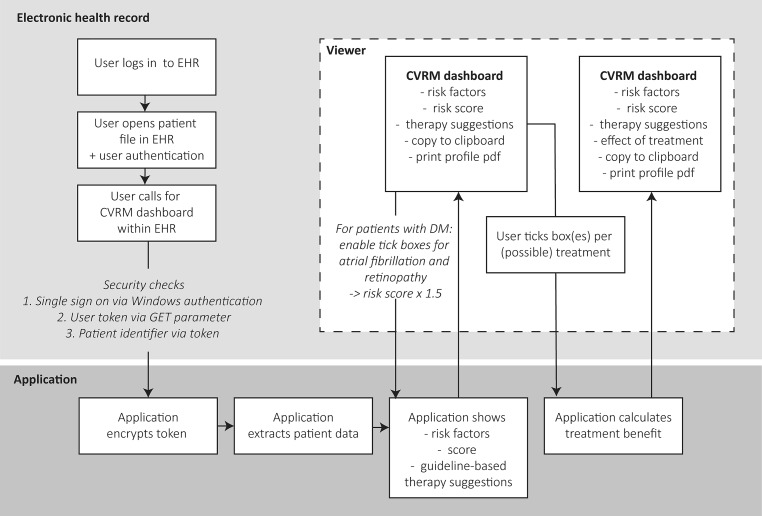
Technical infrastructure. *CVRM* cardiovascular risk management, *EHR* electronic health record, *DM* diabetes mellitus

The practical use of the CDSS is described in Fig. 1. First, a user logs in to the EHR and opens the patient record after EHR-based authentication. By clicking on a link within the EHR, the CDSS is activated. The connection between the data within the EHR and application is established via a URL. Prior to authorising access three security checks are conducted: a single sign-on Windows authentication, user authentication via GET parameters (which guarantees that this user can access this patient data) and a patient identifier via an encrypted token.

Data on the cardiovascular risk profile are extracted from the EHR. Four structured query language (SQL) queries are used to obtain necessary information from the EHR. These four queries are aimed at questionnaire information, measurements, laboratory results, and the medication register.

During the development we used a data lake, a repository of the required anonymised data in its natural format and with enough data storage space and adequate processing speed to develop the CDSS. After the developmental phase and tests on accuracy and processing burden of the queries on the EHR, the dashboard was linked directly to the back end of the EHR, which made data storage within the data lake unnecessary. Our application calculated risk scores and provided suggestions for therapy based on predefined business rules. The application returned the complete dashboard via a viewer. When treatment suggestions were made, these were accompanied by clickable boxes that showed the risk score with or without the proposed treatment. The application calculated the risk modification due to the intervention and returned these data to the viewer via an application program interface.

The expert group decided on essentials and preferences regarding functionalities and user interface.

The user interface, the part of the CDSS the end-user finally works with, was designed taking user experience, characterised by personalisation, accessibility, compatibility and overall intuitiveness into account [10]. The expert group discussed the final interface layout, taking into consideration that personalisation of a tool increases uptake amongst end-users, but decreases scalability. Patient preferences were studied by a patient panel (*n* = 67).

### Data quality and study population

In many CDSSs, data obtained during routine clinical practice are the main source of input data. Clinical data are generated by caregivers in the EHRs but are not primarily intended for further use in either research or feedback. CDSS quality depends on the ‘raw’ data and so ‘garbage in—garbage out’ illustrates the problem well [11].

The Centre for Circulatory Health of the University Medical Centre (UMC) Utrecht initiated the Utrecht Cardiovascular Cohort (UCC) [12]. All first-time patients visiting one of the departments of the Centre for Circulatory Health at the UMC Utrecht for the evaluation of a symptomatic vascular disease or an asymptomatic vascular condition are invited to participate. Information on the cardiovascular risk factors data set, based on Dutch CVRM Guidelines, is collected from all patients at all departments and registered in a structured format in the EHR. The detailed methodology has been published elsewhere [12]. All data used for development of our CDSS were extracted from the files of patients who gave informed consent for research including the use of their routine clinical care data.

### Validation

During the dashboard development phase validation studies were conducted in a test environment, i.e. a copy of the EHRs. Validation included data completeness (i.e. defined as the proportion of information provided versus information needed) and data accuracy (i.e. defined as agreement between the value presented in the dashboard and the ‘raw’ value in the EHR) [11, 13]. These parameters were assessed for risk factor levels, risk estimates, and medication prescription. In addition, checks were performed on the suggested treatment approach given the risk factor levels and risk estimates following the 2016 CVRM guideline, and the estimates of effect when completely adherent to the suggested treatment approach (e.g. the estimated absolute risk reduction when smoking was stopped) [1, 14]. This process was repeated until the accuracy was 100%.

### Evaluation

After validation of the CVRM dashboard prototype, access was given for ‘live’ CDSS use, within the EHR environment, to a limited number of clinicians from inside and outside the expert group (*n* = 7) for feedback on performance and usability. No formal instructions were given, apart from using the prototype in routine care, and no formal itemised questionnaire was used.

## Results

**Fig. 2:**
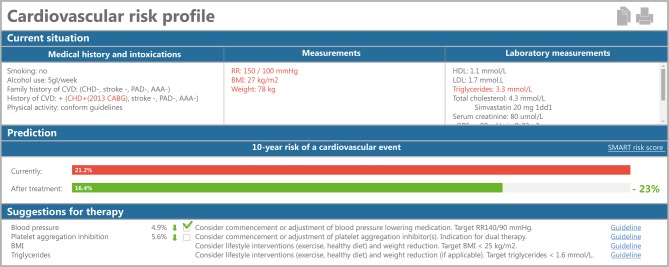
Cardiovascular risk management dashboard in the electronic health record. For a detailed description, please see the Results section

Fig. 2 shows the prototype of the CVRM dashboard presented to the treating physician in the EHR during patient visits to the outpatient department. The CVRM dashboard consists of three parts: risk factors, risk score, and guideline-adherent treatment strategies. The cardiovascular risk factors were displayed together with prescribed medications. For example, if lipid-lowering medication was prescribed, this was displayed in the dashboard below the LDL cholesterol value.

The completeness of all required risk factors was between 88% and 100% for those individuals that were included in the UCC and who gave written informed consent. Information on medications was extracted from the electronic prescription system. The completeness and accuracy of medication registrations was the responsibility of the treating clinician.

Ten-year cardiovascular risk estimates were based on HEART score [1] for those free from previous CVD, the SMART risk score [9] for those with a previous cardiovascular event, the Elderly risk score [15] for those aged 70 years or above, and the ADVANCE risk score [16] for individuals with diabetes mellitus and no previous CVD. Some information needed for the risk prediction algorithms (e.g. hsCRP for SMART and urine protein for ADVANCE) was missing. We used mean imputation to complete the data.

The usability group suggested showing red and green bars for the 10-year estimated cardiovascular risks since the use of common abbreviations and features (such as red for too high values, green for appropriate values) decreases user time and increases efficiency and usability.

The seven physicians that were given access for use of the prototype were enthusiastic but critical. Positive aspects were the one-click-away overview at a glance, the personalised risk score, suggestions for treatment strategy, and the possibility to make a ‘live’ prediction of the estimated benefit of treatment. Yet, when information on risk factors was not collected or recorded in other parts of the EHR than anticipated by the queries, values were missing, and consecutive calculation of risk estimates and treatment suggestions failed. This was particular apparent when patients were not participating in the UCC. This calls for further improvement of the dashboard, specifically in the case of missing data, before widespread implementation and evaluation of efficacy. We suggest efficacy can best be evaluated in a multicentre, prospective, stepped wedge, cluster randomised trial. This design combines elements of a standard parallel cluster randomised design (the intervention is applied in clusters) and a before—after design (each cluster switches to the intervention).

Since CVRM is mostly performed by general practitioners in the Netherlands, the technical design of our application is suitable for embedding in all kinds of EHR environments, including those of general practitioners. The next steps are to test our proof-of-concept application in these environments.

## Discussion

### Summary

We set out to develop a CDSS for use in routine clinical practice in order to improve adherence to CVRM guidelines with the ultimate aim of improving patient healthcare. The planning and development started with writing a project plan to define the scope and requirements with a dedicated multidisciplinary team. This was followed by data work-up, including quality checks, development of methods to improve data completeness, and the writing of business rules to incorporate individual variables and models into decision trees. The technical design was then developed, incorporating aspects of user experience and interface design, ICT architecture and programming. Finally, implementation involved consideration of factors that influence embedding, ICT support and maintenance, sustainability, generation of scientific support, and end-user testing. Based on end-user testing, improvement of the prototype is currently ongoing.

### Challenges: missing data and text

A CDSS uses data collected in routine clinical practice. Whereas most imaging, laboratory, and several other test result data are structurally placed in predefined locations in the EHR, clinical notes are often unstructured [17]. This is an important potential source of inaccurate and/or missing data. For example, systolic pressure is found in several different fields in the EHR. This can either be solved at the source (involving many people in a hospital, calls for education, training, and feedback) or by using search-engine approaches that extract these measurements [10].

In medical practice clinical notes, usually free text, are still the cornerstones of medical reporting, as these allow flexible registration of clinical reasoning with case nuances, and the procedure is less time-consuming [18]. Unstructured text is even worse than unstructured numerical data, such as blood pressure. Spelling errors, domain-specific abbreviations, and idiosyncrasies in a complex context make free text very difficult to analyse computationally. Although text-mining algorithms are available, extensive efforts go into that approach before accurate and complete data are retrieved. Although general text-mining algorithms are available for use, the approach taken should be tailored to the specific situation at each hospital, which hampers scalability.

Missing data, irrespective of its underlying cause, prohibits use of risk-prediction algorithms. Right now, in the worst case with no data, we can impute missing data parameters with the mean from a training data set and generate the average population estimate. But this could cause a bias towards the population average. More advanced statistical approaches for imputations are readily available, but the models for imputations on an individual patient level have to be developed, validated and then integrated in the dashboard approach such that in the end the dashboard nearly always works in routine clinical practice (unless there is too much missing data), provides valid and adequate information, and also informs the treating physician which information was missing and was estimated using statistical modelling. Presently, within the CVRM dashboard project we are exploring imputation and machine-learning approaches to arrive at flexible prediction algorithms and compare the performance of the obtained models with respect to accuracy and impact on clinical decision-making [19].

### Prediction rules

Traditionally, prediction rules on cardiovascular events provide 10-year risk estimates. Yet, risk-factor interventions usually have a lifelong perspective rather than a 10-year horizon. Lifetime benefit seems a more intuitive and understandable measure. Recently, it has become possible to estimate lifetime risks and individual treatment benefits with externally validated risk scores for patients with established CVD (SMART-REACH risk score) and for patients with diabetes mellitus (DIAL score). These scores, as well as 10-year risk scores, are available at the U‑Prevent website (http://www.U-Prevent.com) [9, 15, 20–23].

Our approach to the development of a CDSS facilitates the use of any other prediction rules, e.g. prediction of 30-day mortality, 1‑year mortality, or the balance between risk of haemorrhage and reduction of ischaemic events.

### Clinical impact

Although it is plausible that an appropriate CDSS leads to improved adherence to guidelines and improved patient care, evidence of clinical benefit in this regard seems modest. A recent systematic review by Groenhof and co-workers reported on 22 studies, almost all conducted in primary care [24]. We concluded that CDSSs are related to some improvement in CVRM, but heterogeneity limits overall conclusions. Since the complexity and application of CDSSs can vary greatly, there is no one-size-fits-all approach in testing safety and effectiveness[24].

Moreover, alternative design considerations need to be taken into account for evaluation. Because of the risk of contamination in a classical randomised control trial (RCT) with patients as the randomisation unit, a cluster randomised trial seems the most common design in CDSS studies. But sample size calculations need to allow for intra-cluster correlations. Also, with heterogeneous clusters, statistical performance can be lower. A stepped-wedge cluster RCT could be a solution to this problem: this involves random and sequential cross-over of clusters from control to intervention until all clusters are exposed [25].

Lastly, other outcomes such as patient experiences—as patient empowerment was positively associated with CDSS effect—and reasons as to why clinicians deviate from the guidelines should be evaluated [24, 26].

### Rules and regulations

The rapid developments in information technology also need to be addressed from legal and ethical perspectives. Currently, CDSSs are defined as a medical device in the medical device requirements (MDR) of the European Union [27]. The MDR is the prelude to the Medical Device Directive that will come into effect in 2020, indicating that medical software like a CDSS requires CE certification. The criteria for CE certification of a CDSS are multi-interpretable, but tools that provide a diagnosis, prognosis or therapy advice usually require CE certification. CE-certification processes are, however, only minimally adapted to evaluate devices such as CDSS, characterised by a combination of software, medical evidence (risk scores, other models) and medical practice. Guidance, starting with the developmental phase, by an organ specialising in CE certification, seems appropriate.

### Future potential

In general, CDSSs can be considered as a component of the clinical infrastructure integrating care, data analytics, scientific evidence, and clinical best practice (guidelines) [5].

This project showed a proof of concept for application of data analytics in clinical care. More advanced data analytics techniques such as probabilistic data integrity checks to automatically determine erroneous data and machine learning, potentially improving predictions by learning from complex and non-linear interactions, and previously latent auxiliary variables [28]. Also, decision-tree formulations may be suboptimal because they may assume unrealistically simple situations, whereas in practice patients have multiple problems and treatments might be based on multiple guidelines [2, 29]. More flexible decision guidance, including mitigation frameworks to allow for concurrent processing of multiple guidelines and Markov random field methods are potential solutions to this problem [29, 30]. Practical implementation and adoption of such approaches should first be explored.

If accessible for evaluations, CDSSs can provide data for evaluation of care, including benchmarking of subgroups of clinicians. Furthermore, a CDSS provides the opportunity to identify eligible patients for scientific studies, including pragmatic trials. In this way, CDSSs contribute to development of a learning healthcare system: a healthcare system where care and science are connected via a continuous cycle of data collection in care, data analytics and evaluation, interpretation and implementation [31].

## Conclusion

Development of a CDSS is complex and requires a multidisciplinary approach. We identified opportunities and challenges regarding scope and requirements, infrastructure and interface, data quality and population, and validity. The regulatory environment, including guidance on scientific evaluation, legislation, and privacy issues needs to evolve within this emerging field of eHealth.
